# The Importance of Genomic Literacy and Education in Nursing

**DOI:** 10.3389/fgene.2021.759950

**Published:** 2021-12-14

**Authors:** Dijana Majstorović, Anita Barišić, Mauro Štifanić, Igor Dobrača, Jadranka Vraneković

**Affiliations:** ^1^ Faculty of Medicine, Juraj Dobrila University of Pula, Pula, Croatia; ^2^ Department of Medical Biology and Genetics, Faculty of Medicine, University of Rijeka, Rijeka, Croatia; ^3^ Faculty of Natural Science, Juraj Dobrila University of Pula, Pula, Croatia

**Keywords:** curriculum, education, genomics, literacy, nursing

## Abstract

Genetic discoveries and technological advances have been changing nursing care delivery, which modifies the roles and practices of nursing in society. Although the need for education of nurses in the field of genomics has been recognized in the 1960s, many countries still have no clear guidelines in this field of education and training. The purpose of this study was to evaluate current genomics content in the curriculum of undergraduate and graduate programs of studies in nursing in Croatia, and to measure the genomic literacy of Croatian undergraduate nursing students through assessing participants’ understanding of genomic concepts most critical to nursing practice. The curriculum of undergraduate and graduate programs of nursing classes of 2020/2021 were independently analyzed by the authors. For measuring the knowledge of essential genomic concepts among nurses, a Genomic Nursing Concept Inventory (GNCI^©^) instrument was employed. Results indicate that the current genomics content, for undergraduate and graduate nursing programs in Croatia, is inadequate and not concordant among universities. Moreover, the genomic literacy of Croatian undergraduate students (Undergraduate program 10) was found to be low. Scores across respondents ranged from 3 to 22 (out of possible 31), with a mean scale score 9.8 (SD 5.3) (31.6% correct). We can conclude that the curriculum for undergraduate and graduate programs of Studies in nursing should be revised to implement the latest genomic practices and approaches to genomics education while nurses should acquire an adequate level of genomic literacy in order to produce desired outcomes of competency in nursing practice.

## Introduction

Development in genomics and its implementation in the healthcare system worldwide has been steadily increasing (Skirton et al., 2010; Murakami et al., 2020). Moreover, genomic knowledge has been applied in different areas: promotion of health, prevention of injuries and diseases, diagnostics, therapy, patient counseling, support and education (International Society of Nurses in Genetics, 2020). Nurses, as the largest professional group in healthcare system and as a primary contact with patients, have a significant role in the interpretation of genomic data relevant to the care of the patient (World Health Organization, 2016). However, international studies demonstrate a lack of nurses’ genomic literacy, and confidence in applying this information to their work (Skirton et al., 2012; Godino et al., 2013; Umberger et al., 2013; Donnelly et al., 2017; Read and Ward, 2018; Wright et al., 2018). Although the urgency for education of nurses in the field of genetics was recognized in the early 1960s (Brantl and Esslinger, 1962), many countries, including Croatia, still have no clear guidelines in aforementioned field of education and training (Mc Cormick and Calzone, 2017). This is evident throughout the insufficiently represented genomic curriculum contents (Prows et al., 2005; Giddens and Brady, 2007; Collins and Stiles, 2011; Daack-Hirsch et al., 2012; Giarelli and Reiff, 2012; Calzone et al., 2014; Camak, 2016).

In Croatia, the education of nurses at the academic level is organized at the undergraduate and graduate studies. The harmonization of undergraduate studies is prescribed by the Common compulsory part of the undergraduate study program of nursing (core curriculum) (Ministry of Science and Education Republic of Croatia, 2014). This document is in line with the provisions of Directive 2005/36/EC (EUR-Lex, 2020). Core curriculum prescribes 47 compulsory courses during 3 years [158 European Credit Transfer and Accumulation System (ECTS)] in the field of nursing care, basic and social sciences. These courses constitute 87.7% of the program, whereas the remaining 22 ECTS credits are designed by each higher education institution. Nurses with a Bachelor degree learn to independently determine individuals basic human needs, plan, implement, record, and evaluate health care, participate in diagnostic and therapeutic procedures and conduct education. The graduate-level program lasts 2 years (120 ECTS) and is designed exclusively by the higher education institution that runs the program. Nurses with a Master’s degree work at all levels of health and social care, independently plan and organize all work processes in the field of nursing care, participate in research processes and complex diagnostic and therapeutic procedures. Moreover, the Proposal of the Standard of Occupations, the Croatian Classification Framework, prescribes the key tasks and competencies of the Master of Nursing. The Framework emphasizes that implementing evidence-based knowledge is the key to successful nursing practice, as well as conducting research on the latest advances in healthcare, which may include genomics (Croatian Qualifications Framework, 2017).

In order to assess the readiness for integrating genomics into nursing curriculum in Croatia, we conducted a survey study with two specific aims: 1) to evaluate the current genomics content in both undergraduate and graduate nursing programs in Croatia (academic year 2020/2021), and 2) to measure the genomic literacy of Croatian undergraduate nursing students through assessing their understanding of core concepts in genomics which are most critical to nursing care.

## Materials and Methods

### Evaluation of the Current Genomics Content in the Curriculum

The authors first collected curriculums of all compulsory, additional and elective courses in undergraduate and graduate programs, reviewed them, and determined how many of those curriculums included a genomic component. Undergraduate programs were explored through the National Information System for Application to Higher Education Institutions (National Information System for Application to Higher Education Institutions, 2021 ; Working group, 2013), and graduate programs through the university websites (Supplementary Material S1). The types of courses in the undergraduate study of nursing are divided into compulsory courses which are prescribed by the core curriculum (47 courses, 158 ECTS) and additional courses (22 ECTS) which are designed by the university. Additional courses can be organized as compulsory and elective courses.

### Measuring the Genomic Literacy of Croatian Undergraduate Nursing Students

#### Study Design

The anonymous online survey was conducted over 2-month period (March 2021–May 2021). It was approved by the Institutional Review Board of Undergraduate program 10. Inclusion criteria were students attending the Undergraduate nursing program 10 (1st–3rd year), Faculty of Medicine at the time of the survey administration and their willingness to participate in the study. There were no age or sex restrictions for participation.

#### Instrument

Survey data were collected using an online open-source software survey program Limesurvey. It consisted of demographic data shown in Table 1 and the 31 multiple-choice questions that constitute the Genomic Nursing Concept Inventory (GNCI^©^) (Ward, 2011). The GNCI^©^ is an internationally validated questionnaire developed for assessment of genomic literacy among nurses across four topical categories (Genome Basics, Mutations, Inheritance Patterns, Genomic Healthcare) and eighteen foundational genomic concepts (Ward, 2011; Ward et al., 2014; Ward et al., 2016a). Permission to use the instrument was obtained from the author of the GNCI^©^. The survey was translated into the Croatian language.

**TABLE 1:** Characteristics of the participants (*n* = 53).

Variable	N (%)
Gender	
Male	6 (11.3)
Female	47 (88.7)
Employment	
Yes	10 (18.9)
No	43 (81.1)
Number of years in nursing	
0	43 (81.1)
1–5	4 (7.5)
6–10	3 (5.7)
>10	3 (5.7)
Acquired knowledge about genomics through	
Literature on genomic topics	24 (45.3)
Previous genomic course	3 (5.7)
Previous genomic workshop	1 (1.9)
Other resources	27 (50.9)
Curricular progression	
1st year	39 (73.6)
2nd year	8 (15.1)
3rd year	6 (11.3)

#### Data Analysis

Data were analyzed using SPSS^®^ version 25 software (IBM Corp., Armonk, NY, United States). Descriptive statistics were used to determine trends in the demographic data. Each of the 31 GNCI^©^ questions was scored as correct or incorrect and a total number of correct answers (range 0–31) was calculated for each participant. Mean scores for the four topical categories were calculated using a specified item data in each category.

## Results

### Evaluation of the Current Genomics Content in the Curriculum

Table 2 shows the core curriculum for undergraduate studies (academic year 2020/2021) in Croatia. Ten universities in Croatia provide either full–time or part–time undergraduate study program, which enrolled 899 students in academic year 2020/2021. The nominal distribution among compulsory, additional and elective courses was similar between universities (Table 2). Genomics is included as a part of subject at each university, mainly throughout the Pediatrics course, with no defined learning outcomes and a number of hours related to genetic content. Only two undergraduate nursing programs (Undergraduate programs 5 and 10) provide independent genomic courses for Bachelor of nursing students.

**TABLE 2:** Curriculum for Undergraduate studies in nursing (academic year 2020/2021) in Croatia.

University		Enrollment quota		Courses				
		Full–time study program	Part–time study program	Compulsory	Additional		Genomics included as	
					Compulsory	Elective	Part of subject*	Independent subject
1	Undergraduate program 1	95	30	48	1	23	+	-
2	Undergraduate program 2	30	80	54	7	42	+	-
3	Undergraduate program 3	30	120	48	2	16	+	-
4	Undergraduate program 4	30	0	58	12	8	+	-
5	Undergraduate program 5	0	21	49	2	18	+	+
6	Undergraduate program 6	42	60	47	0	25	+	-
7	Undergraduate program 7	41	20	50	3	10	+	-
8	Undergraduate program 8	47	0	51	4	9	+	-
9	Undergraduate program 9	33	117	47	0	31	+	-
10	Undergraduate program 10	73	30	47	0	9	+	+
Total		421	478	499	31	191		

*No defined learning outcomes and a number of hours related to genetic content.

Table 3 shows the curriculum for graduate studies (academic year 2020/2021) in Croatia. Nine universities in Croatia provide either full–time or part–time program, which enrolled 504 students in academic year 2020/2021. The nominal distribution among compulsory and elective courses vary a lot between universities (Table 3). Genomics modules are taught as part of other subject-matter courses (Table 3). Graduate programs 6 and 9 do not integrate any genomics content in their nursing curriculum. Curricula contents were not available for all courses at the Graduate programs 4 and 7. Only two graduate nursing programs (Graduate programs 5 and 8) provide independent genomic courses for Master of nursing students.

**TABLE 3:** Curriculum for Graduate studies in nursing (academic year 2020/2021) in Croatia.

University		Enrollment quota		Courses			
		Full–time study program	Part–time study program	Compulsory	Elective	Genomics included as	
						Part of subject*	Independent subject
1	Graduate program 1	0	20	110	60	+	-
2	Graduate program 2	0	40	50	57	+	-
3	Graduate program 3	30	150	13	10	+	-
4	Graduate program 4	0	20	25	5	-	-
5	Graduate program 5*	0	0	32	11	+	+
6	Graduate program 6	28	46	16	19	-	-
7	Graduate program 7	15	15	15	18	-	-
8	Graduate program 8	20	70	20	13	+	+
9	Graduate program 9	20	30	9	13	-	-
Total		113	391	290	206		

*No defined learning outcomes and a number of hours related to genetic content; **academic year 2020/2021 not enrolled.

### Measuring the Genomic Literacy of Croatian Undergraduate Nursing Students

Initially, 189 students were invited to participate in the study, whereas 108 students clicked on the survey link. Sixty-eight inventories had missing responses to one or more test items. Those were excluded from analysis, which resulted in a final sample of 53 students. Ages ranged from 19 to 40 years (median 23.5 years). Most respondents identified as female (88.7%), were currently unemployed (81.1%), reported to be in the first year of their studies (73.6%) and acquired knowledge in genomics primarily by reading the literature (Table 1).

For each participant, a total number of correct answers (range 0–31) was calculated. Scores across all respondents ranged from 3 to 22, with a mean scale score of 9.8 (SD 5.3) (31.6% correct). Topical category scores were highest on “Inheritance” (33.2%), and lowest on “Mutations” (19.5%). A description of each item, along with the number of correct answers and the mean score of topical category is provided in Table 4. In relation to questions, student scores were highest in response to DNA sequence (Question 2, 69.8% answered correctly). In contrast, lowest scores were in distinguishing germline and somatic mutations (Question 18, 3.8% answered correctly).

**TABLE 4:** GNCI^©^ item scores, topical category scores and total score.

Topical category	Domain	Item	Concept	Number of correct answers (%)
Genome Basics	Genome composition/ organization	2	DNA sequence = the order of nucleotides	37 (69.8)
		4	All cells contain an entire set of genes	5 (9.4)
		5	Genome organization (amount of DNA in the human genome)	21 (39.6)
		8	Organization of DNA (genome-chromosome-gene nucleotide)	6 (11.3)
	Homo and heterozygosity	13	Heterozygosity = two functionally different gene alleles	4 (7.5)
		29	People with AD diseases are usually heterozygous for the mutation	15 (28.3)
	Gene function/ expression	1	Gene function/expression	7 (13.2)
		6	Central dogma (product of DNA transcription-translation = protein)	9 (17.0)
		9	Specific role of a gene in determining a trait (produces a protein)	10 (18.9)
		11	Nature of gene expression (distinct from gene sequence)	13 (24.5)
	Genotype-phenotype association	7	Meaning of ‘genotype’ (distinguished from phenotype)	15 (28.3)
	Human genome variation	3	>99% of DNA sequence of unrelated people is identical	8 (15.1)
		20	All people have the same set of genes (e.g., BRCA)	11 (20.8)
	**Mean score of topical category**			**12.4 (SD = 8.8)**
Mutations	Mutations and disease	19	Genetic heterogeneity (people with the same genetic condition may have unique mutations)	14 (26.4)
		21	DNA alterations cause disease by altering protein production	15 (28.3)
	Germline and somatic mutations	18	Distinguishing germline and somatic mutations	2 (3.8)
	**Mean score of topical category**			**10.3 (SD = 7.2)**
Inheritance	Dominance	10	The meaning of dominance	13 (24.5)
	Autosomal inheritance	24	Autosomal disorders are inherited equally by males and females	14 (26.4)
	Autosomal dominant inheritance	30	Calculating inheritance risk in AD disease	23 (43.4)
		31	Inheritance risk is fixed and independent of number of offspring	20 (37.7)
	Autosomal recessive inheritance	15	Parents of offspring with AR conditions are obligate carriers	14 (26.4)
		16	Calculating inheritance risk in AR disease	25 (47.2)
	X-linked inheritance	17	Understanding inheritance of X-linked disorders	20 (37.7)
	Multifactorial inheritance	25	Multifactorial etiology of complex diseases	12 (22.6)
	**Mean score of topical category**			**17.6 (SD = 4.5)**
Genomic healthcare	Family health history	23	Identifying red flags (risk factors)	10 (18.9)
		26	Utility of family history to predict risk for complex disease	17 (32.1)
	Pharmacogenomics	12	Mutations can cause people to respond unpredictably to drugs	34 (64.2)
		27	A drug receptor is a protein (genetics and pharmacodynamics)	15 (28.3)
		28	Genes influence drug response via their effect on proteins	11 (20.8)
	Genetic testing	14	Meaning of a positive screening test	27 (50.9)
		22	Purpose of carrier testing	8 (15.1)
	**Mean score of topical category**			**17.4 (SD = 9.6)**
**Mean total score**				**14.7 (SD = 8.2)**

## Discussion

### Evaluation of the Current Genomics Content in the Core Curriculum

This study represents the first evaluation of the current genomics content in the curriculum of undergraduate and graduate program of Studies in nursing in Croatia (academic year 2020/2021). Data were derived from the curriculum of ten undergraduate and nine graduate nursing programs. Our findings show that the genomics is marginalized in the education of nurses, both at the undergraduate and graduate levels.

Our findings corroborate that genomics curriculum is less developed at the undergraduate than the graduate level likely because graduate programs are mandated by the Ministry of Education (Ministry of Science and Education Republic of Croatia, 2014) to include genomics content to comply with European Directive 2005/36/EC) (EUR-Lex, 2020). The Directive prescribes in detail the minimum requirements for a 3-year education of nurses, 4,600 h of theoretical and clinical training, and compulsory courses in the field of nursing care, basic and social sciences. Representatives of all higher education institutions conducting undergraduate nursing study programs in Croatia organized in 2013 a working group for the harmonization of nursing study programs with the provisions of Directive 2005/36/EC. Besides, the working group has developed a core curriculum that defines each compulsory course at the undergraduate level (Working group, Ministry of Science and Education Republic of Croatia, 2014). During the development of the core curriculum, the working group did not follow the results of numerous studies showing a low level of genomic knowledge (Bankhead et al., 2001; Bottorf et al., 2005; Maradiegue et al., 2005; Tomatir et al., 2006). Neither recommendations of The European Society of Human Genetics (ESHG) to facilitate the development of genomic health care in the European community were adopted into the core curriculum (The European Society of Human Genetics, 2008). A review of the directive shows that genomics is only mentioned in the education study program for veterinary surgeons as part of the specific subjects related to basic science (EUR-Lex, 2020). As the significance of genomics in the education of nurses was not recognized, the opportunity to incorporate basic competencies in genomics (The European Society of Human Genetics, 2008) into the core curriculum was missed. However, curricula of the Undergraduate programs 5 and 10, include genomics both, as a part of other subject and as an independent subject. In all other universities’ curricula, genomics is present only as a part of compulsory courses. No expected learning outcomes are specified nor are guidance about the number of hours genomics should occupy in the total course schedule. At the graduate level, the situation is similar. Among nine universities, only Graduate programs 5 and 8 offer genomics both as part of the subject and independent subject.

Although the Undergraduate programs 5 and 10, as well as graduate programs 5 and 8, represent Croatian Universities that recognize the importance of genomic education among nurses, when compared to examples of good practice at the international level (e.g., The University of Texas Permian Basin, 2020), their programs are still insufficient. Namely, outcomes at the international level include higher levels of knowledge, which enables skills acquisition and the development of attitudes, content is focused on incorporating genomics knowledge and technologies in nursing practice, obligatory and recommended materials are from the field of nursing practice, and the curriculum is entirely based on established competencies.

### Measuring the Genomic Literacy of Croatian Undergraduate Nursing Students

Our results indicate that the genomic literacy of Croatian undergraduate nursing students (Undergraduate program 10) is low. Interestingly, the majority of participants that initially clicked the survey link gave up during the survey, because, as stated in their reasons, “it was too difficult.” This could signal a genomic knowledge deficit among the students we recruited to survey. The reason for this can perhaps be found in the fact that most of the participants were first-year students, and did not attend any genomic course that may improve their understanding of genomics (e.g., compulsory Pediatrics course or an elective course Human Genetic Disease).

With a mean total score of 9.8 (SD 5.3; score range 3–22; 31.6% correct answers), our results are marginally lower in comparison to the results of other studies that utilized the GNCI^©^ instrument. Namely, Wright et al. reported a mean score of 13.3 (SD 4.60; score range 3–29) (Wright et al., 2019), McCabe et al. 13.7 (SD 4.9; score range 5–26; 44% correct answers) (McCabe et al., 2016), while Ward et al., reported a mean score of 12.85 (SD 4.64; score range 3–28; 41.5% correct responses) (Ward et al., 2016b), and finally, Read and Ward published 14.9 (SD 5.3; score range 4–31; 48% correct answers) (Read and Ward, 2016). To reflect on the results of the other studies that used different methods for genomic literacy assessment, the integrative reviews by Wright et al., and Godino et al., were analyzed (Godino et al., 2013; Wright et al., 2018). The reported conclusions of these studies are similar, declaring inadequate genomic competency among nurses, which closely correlates with a level of their genomic literacy. The authors underline the importance of assessing actual content knowledge using a validated inventory instrument e.g., GNCI rather than self-reports to best capture genomic literacy in future studies. However, considering motivation as a very important factor in the learning process (Ferreira et al., 2011), the estimation of self-reported motivation and/or perceived knowledge should also be included.

## Limitations

The results of this study should be interpreted in light of several limitations. Namely, curricula contents were not available for all courses at the websites of all universities (Graduate programs 4 and 7), while the available curricula were not concordant among universities. Furthermore, the curricula analyzed either did not provide information on the number of hours devoted to genomics or if listed, did not specify how student knowledge was assessed. Measuring the genomic literacy of Croatian undergraduate nursing students was limited by the Covid-19 pandemic. Online impersonal assessment made it difficult to motivate students and ended with poor recruitment and participation of students. It resulted in small sample size, mostly consisting of first-year students, from a single nursing program. In addition, the participation of second and third-year students in small numbers prevented the previously planned comparison of knowledge between students of different years of study. Nonetheless, although the survey response rate was lower than expected, our study findings provide a valuable snapshot of genomic literacy among under/graduate nursing students.

## Final Recommendations and Conclusion

Based on the results of our research, it is necessary to create the preconditions for efficient and effective implementation of genomics in the education of nurses in Croatia. The inclusion of genomics should be harmonized throughout the curriculum, with well-defined outcomes that should be coordinated with international documents prescribing the competencies of nurses in the field of genomics, respecting the specifics of each country. Existing curricula should be reviewed to ensure only genomics concepts that are relevant to nursing practice are added to improve literacy. Moreover, the assessment of genomic literacy throughout the validated questionnaires should be accomplished before the development of the curriculum so that course content can be directed to knowledge deficits. Furthermore, our recommendation would be to implement the genomic content from the beginning of the studies in order to adopt the basic knowledge, which should be upgraded during continuous education. The genomic content at the graduate level should be harmonized with the outcomes that students acquire upon completion of the study program. Besides, elective genomic courses should be offered to students who want to acquire more knowledge and skills related to genomics. Since it is essential to weave genomics throughout the curriculum, it is necessary to support teachers in this area. We must not forget the nurses who completed their education according to curricula that did not include genomic content. Multifactorial barriers to increasing the amount of genomics content in nursing curricula include: insufficient genomics knowledge base of most nursing faculty, limited numbers of faculty in various programs who view genomics as relevant to nursing practice, perceived inability to add more content to an already crowded curriculum, and a lack of regulatory agencies of nursing requiring competency in genomics as part of the licensure.

Due to the fact that research is carried out with a small student population, mostly in their first year of undergraduate, our plan would be to replicate the research on the national level, which means recruiting each University in Croatia that provides a nursing Program, using the GNCI^©^ questionnaire, which would allow scaling up the results with adequate sample size. Moreover, this would provide insight into genomic literacy among Croatian nursing students and serve as a good starting point for the development of a nationally adapted curriculum.

Overall, more must be done to ensure that Croatian nurses have an adequate level of genomic literacy (knowledge of the role of the genomic factors in health and disease, understanding of the utility and limitations of genomic testing and information, upholding the rights of all individuals to informed decision making) to deliver optimal nursing care. Study programs have to be in line with modern clinical nursing practice, which definitely require the implementation of genomics into the curriculum, either as a part of a compulsory course or/and a separate one. Also, we believe that mandatory non-formal education, prescribed by the Croatian Chamber of Nurses is a good ground for the inclusion of genomic content as well. Finally, genomics is increasingly important in all areas of clinical nursing practice, and the effectiveness of courses and curricula in developing genomic competence among students is of high priority for evolving modern nursing practice.

## Data Availability

The raw data supporting the conclusions of this article will be made available by the authors, without undue reservation.
