# Supplementary material for: The Importance of Genomic Literacy and Education in Nursing
Source: Front Genet. 2021 Dec 14;12:759950. doi: 10.3389/fgene.2021.759950 (PMC8712715; doi:10.3389/fgene.2021.759950)
Supplement: Supplementary file 1 [file Table1.docx]

Supplementary Material

# Supplementary material 1. List of undergraduate and graduate studies in nursing and links to their websites and study programs.

|  | **Program (link to the website)** | **Link(s) to the study programs** | **References** |
| --- | --- | --- | --- |
| 1 | Undergraduate program 1  Graduate program 1  ([University of Applied Health Sciences, Zagreb, Study Programme in Nursing](https://www.zvu.hr/?lang=HR)) | [Professional Study Program in Nursing](https://www.zvu.hr/static/np/npip/15.html); [Specialist Graduate Professional Study in Clinical Nursing;](https://www.zvu.hr/static/np/npip/50.html) [Specialist Graduate Professional Study – Public Health;](https://www.zvu.hr/static/np/npip/18.html) [Specialist Graduate Professional Study in Psychiatric Nursing;](https://www.zvu.hr/static/np/npip/19.html)  [Specialist Graduate Professional study of “Management in Nursing”](https://www.zvu.hr/static/np/npip/17.html) | University of Applied Health Sciences Zagreb, Study Programme in Nursing. (2021). <https://www.zvu.hr/?lang=HR> [Accessed March 1, 2021].  University of Applied Health Sciences Zagreb, Study Programme in Nursing. (2021). Professional Study Program in Nursing Curriculum academic year 2020/2021. <https://www.zvu.hr/static/np/npip/15.html> [Accessed March 15, 2021].  University of Applied Health Sciences Zagreb, Study Programme in Nursing. (2021) Specialist Graduate Professional Study in Clinical Nursing Curriculum academic year 2020/2021. <https://www.zvu.hr/static/np/npip/50.html> [Accessed March 15, 2021].  University of Applied Health Sciences Zagreb, Study Programme in Nursing. (2021). Specialist Graduate Professional Study – Public Health Curriculum academic year 2020/2021. <https://www.zvu.hr/static/np/npip/18.html> [Accessed March 15, 2021].  University of Applied Health Sciences Zagreb, Study Programme in Nursing. (2021). Specialist Graduate Professional Study in Psychiatric Nursing Curriculum academic year 2020/2021. <https://www.zvu.hr/static/np/npip/19.html> [Accessed March 15, 2021].  University of Applied Health Sciences Zagreb, Study Programme in Nursing. (2021). Specialist Graduate Professional study of “Management in Nursing” Curriculum for the academic year 2020/2021. <https://www.zvu.hr/static/np/npip/17.html> [Accessed March 15, 2021]. |
| 2 | Undergraduate program 2  Graduate program 2  ([The Faculty of Health Studies University of Rijeka, Study Programme in Nursing](https://www.fzsri.uniri.hr/hr/)) | [Professional Study of Nursing](http://www.fzsri.uniri.hr/hr/studenti/nastava/110-preddiplomski-strucni-studiji/sestrinstvo.html); [Graduate University Study of Nursing – Promotion and Protection of Mental Health](http://www.fzsri.uniri.hr/hr/studenti/nastava/117-sveucilisni-diplomski-studiji/sestrinstvo-promicanje-i-zastita-mentalnog-zdravlja.html); [Graduate University Study of Nursing – Healthcare Management](http://www.fzsri.uniri.hr/hr/studenti/nastava/118-sveucilisni-diplomski-studiji/sestrinstvo-menadzment-u-sestrinstvu.html); [Graduate University Study of Clinical Nutrition](http://www.fzsri.uniri.hr/hr/studenti/nastava/150-sveucilisni-diplomski-studiji/klinicki-nutricionizam.html) | The Faculty of Health Studies University of Rijeka, Study Programme in Nursing. (2021). <https://www.fzsri.uniri.hr/hr/> [Accessed March 1, 2021].  The Faculty of Health Studies University of Rijeka, Study Programme in Nursing. (2021). Professional Study of Nursing Curriculum academic year 2020/2021. <http://www.fzsri.uniri.hr/hr/studenti/nastava/110-preddiplomski-strucni-studiji/sestrinstvo.html> [Accessed March 15, 2021].  The Faculty of Health Studies University of Rijeka, Study Programme in Nursing. (2021). Graduate University Study of Nursing – Promotion and Protection of Mental Health Curriculum academic year 2020/2021.<http://www.fzsri.uniri.hr/hr/studenti/nastava/117-sveucilisni-diplomski-studiji/sestrinstvo-promicanje-i-zastita-mentalnog-zdravlja.html> [Accessed March 15, 2021].  The Faculty of Health Studies University of Rijeka, Study Programme in Nursing. (2021). Graduate University Study of Nursing – Healthcare Management Curriculum academic year 2020/2021. <http://www.fzsri.uniri.hr/hr/studenti/nastava/118-sveucilisni-diplomski-studiji/sestrinstvo-menadzment-u-sestrinstvu.html> [Accessed March 15, 2021].  The Faculty of Health Studies University of Rijeka, Study Programme in Nursing. (2021). Graduate University Study of Clinical Nutrition Curriculum academic year 2020/2021. <http://www.fzsri.uniri.hr/hr/studenti/nastava/150-sveucilisni-diplomski-studiji/klinicki-nutricionizam.html> [Accessed March 15, 2021]. |
| 3 | Undergraduate program 3  Graduate program 3  ([The Josip Juraj Strossmayer University of Osijek, Faculty of Dental Medicine and Health Osijek](http://www.fdmz.hr/index.php/hr/)) | [Undergraduate university study programme of Nursing](http://www.fdmz.hr/index.php/hr/studij/preddiplomski-sveucilisni-studij-sestrinstvo); [Graduate university study programme of Nursing](http://www.fdmz.hr/index.php/en/study-programme/graduate-university-study-programme-of-nursing) | The Josip Juraj Strossmayer University of Osijek, Faculty of Dental Medicine and Health Osijek. (2021). <http://www.fdmz.hr/index.php/hr/> [Accessed March 1, 2021].  The Josip Juraj Strossmayer University of Osijek, Faculty of Dental Medicine and Health Osijek. (2021). Undergraduate university study programme of Nursing Curriculum academic year 2020/2021. <http://www.fdmz.hr/index.php/hr/studij/preddiplomski-sveucilisni-studij-sestrinstvo> [Accessed March 15, 2021].  The Josip Juraj Strossmayer University of Osijek, Faculty of Dental Medicine and Health Osijek. (2021). Graduate university study programme of Nursing Curriculum academic year 2020/2021. <http://www.fdmz.hr/index.php/en/study-programme/graduate-university-study-programme-of-nursing> [Accessed March 15, 2021]. |
| 4 | Undergraduate program 4  Graduate program 4  ([University of Split, University Department of Health Studies](http://ozs.unist.hr/studijski-programi/diplomski-sveucilisni-studiji/sestrinstvo)) | [Undergraduate university study programme of Nursing](http://ozs.unist.hr/studijski-programi/preddiplomski-sveucilisni-studiji/sestrinstvo); [Graduate university study programme of Nursing](http://ozs.unist.hr/studijski-programi/diplomski-sveucilisni-studiji/sestrinstvo) | University of Split, University Department of Health Studies. (2021). Undergraduate university study programme of Nursing Curriculum academic year 2020/2021. <http://ozs.unist.hr/studijski-programi/preddiplomski-sveucilisni-studiji/sestrinstvo> [Accessed March 15, 2021].  University of Split, University Department of Health Studies. (2021). Graduate university study programme of Nursing Curriculum academic year 2020/2021. (2021). <http://ozs.unist.hr/studijski-programi/diplomski-sveucilisni-studiji/sestrinstvo> [Accessed March 15, 2021]. |
| 5 | Undergraduate program 5  Graduate program 5  ([University of Dubrovnik, Department of Nursing](https://www.unidu.hr/studij-sestrinstvo/)) | [Undergraduate study programme of Nursing](https://www.unidu.hr/sestrinstvo/#repozitorij); [Graduate Professional Study in Clinical Nursing](https://www.unidu.hr/klinicko-sestrinstvo/#repozitorij) | University of Dubrovnik, Department of Nursing. (2021). <https://www.unidu.hr/studij-sestrinstvo/> [Accessed March 1, 2021].  University of Dubrovnik, Department of Nursing. (2021). Undergraduate study programme of Nursing Curriculum academic year 2020/2021. <https://www.unidu.hr/sestrinstvo/#repozitorij> [Accessed March 15, 2021].  University of Dubrovnik, Department of Nursing. (2021). Graduate Professional Study in Clinical Nursing Curriculum academic year 2020/2021. <https://www.unidu.hr/klinicko-sestrinstvo/#repozitorij> [Accessed March 15, 2021]. |
| 6 | Undergraduate program 6  ([Bjelovar University of applied sciences](https://vub.hr/)) | [Professional Study Nursing](https://vub.hr/strucni-studij-sestrinstvo/) | Bjelovar University of applied sciences. (2021). <https://vub.hr/> [Accessed October 29, 2021]  Bjelovar University of applied sciences. (2021). Professional Study Nursing Curriculum academic year 2020/2021. <https://vub.hr/strucni-studij-sestrinstvo/> [Accessed March 15, 2021]. |
| 7 | Undergraduate program 7  Graduate program 6  ([Catholic University of Croatia, Department of Nursing](http://www.unicath.hr/sestrinstvo)) | [Undergraduate and Graduate university study programme of Nursing](http://unicath.hr/izvedbeni-planovi-20-21/odjel-za-sestrinstvo-arhiva/) | Catholic University of Croatia, Department of Nursing. (2021). <http://www.unicath.hr/sestrinstvo> [Accessed March 1, 2021].  Catholic University of Croatia, Department of Nursing. (2021). Undergraduate and Graduate university study programme of Nursing Curriculum academic year 2020/2021. <http://unicath.hr/izvedbeni-planovi-20-21/odjel-za-sestrinstvo-arhiva/> [Accessed March 15, 2021]. |
| 8 | Undergraduate program 8  Graduate program 7  ([University of Zadar, Department of Health Studies](https://zdravstvo.unizd.hr/)) | [Undergraduate and Graduate university study programme of Nursing](https://zdravstvo.unizd.hr/izvedbeni-plan-nastave/izvedbeni-plan-2020-2021) | University of Zadar, Department of Health Studies. (2021). <https://zdravstvo.unizd.hr/> [Accessed March 1, 2021].  University of Zadar, Department of Health Studies. (2021). Undergraduate and Graduate university study programme of Nursing. <https://zdravstvo.unizd.hr/izvedbeni-plan-nastave/izvedbeni-plan-2020-2021> [Accessed March 15, 2021]. |
| 9 | Undergraduate program 9  Graduate program 8  ([University North, Department of Nursing](https://www.unin.hr/)) | [Undergraduate study of “Nursing”](https://www.unin.hr/sestrinstvo/kolegiji/); [Graduate University Study of Nursing – Healthcare Management](https://www.unin.hr/sestrinstvo/diplomski-sveucilisni-studij/kolegiji/) | University North, Department of Nursing. (2021). <https://www.unin.hr/> [Accessed March 1, 2021].  University North, Department of Nursing. (2021). Undergraduate study of “Nursing” Curriculum academic year 2020/2021. <https://www.unin.hr/sestrinstvo/kolegiji/> [Accessed March 15, 2021].  University North, Department of Nursing. (2021). Graduate University Study of Nursing – Healthcare Managemen Curriculum academic year 2020/2021. <https://www.unin.hr/sestrinstvo/diplomski-sveucilisni-studij/kolegiji/> [Accessed March 15, 2021]. |
| 10 | Undergraduate program 10  ([Juraj Dobrila University of Pula, Faculty of Medicine](https://mfpu.unipu.hr/)) | [Professional Study Program in Nursing](https://mfpu.unipu.hr/mfpu/za_studente/nastavni_planovi) | Juraj Dobrila University of Pula. (2021). <https://www.unipu.hr/> [Accessed October 29, 2021]  Juraj Dobrila University of Pula, Faculty of Medicine. (2021). Professional Study Program in Nursing Curriculum academic year 2020/2021. <https://mfpu.unipu.hr/mfpu/za_studente/nastavni_planovi> [Accessed March 15, 2021]. |
| 11 | Graduate program 9  ([University of Zagreb School of Medicine, University graduate study of nursing](https://mef.unizg.hr/studiji/diplomski/diplomski/)) | [Graduate University Study of Nursing](https://mef.unizg.hr/studiji/diplomski/diplomski/) | University of Zagreb School of Medicine, University graduate study of nursing. (2021). Curriculum academic year 2020/2021. <https://mef.unizg.hr/studiji/diplomski/diplomski/> [Accessed March 1, 2021]. |
